# Surveillance of Injuries in a Tertiary Care Hospital

**DOI:** 10.4103/0970-0218.62572

**Published:** 2010-01

**Authors:** SP Suryanarayana, MS Gautham, Mali Manjunath, V Narendranath

**Affiliations:** Department of Community Medicine, M S Ramaiah Medical College, M S Ramaiah Memorial Hospital, Bangalore - 560 054, Karnataka, India

## Introduction

Injuries are increasingly recognized as a global public health epidemic. Injuries represent 12% of the global burden of disease and third most important cause of overall mortality. Deaths from road traffic injuries account for around 25% of total injury deaths.(1)

A recent national review in India has estimated that a million injury deaths and 30 million hospitalizations occur every year at an average of 10.1 per lakh population. Southern Indian states reported higher number of deaths. 15.4 per lakh injuries occur in Karnataka with Bengaluru city reported 5660 injury deaths with suicides (2429) outscoring RTIs as the leading cause.(2) Largely as a consequence of epidemiological and demographic transitions in recent years, injuries have become a leading cause for morbidity and mortality.

The problem is hidden and unrecognized due to the absence of good quality information within the health and related sectors. Systematic efforts need to be developed urgently in India to address the problem. Information that is relevant, complete, appropriate, timely, and representative is very vital to formulate injury prevention programs.

There is a need to collect data beyond just number of deaths and causes of injuries. Data regarding the host attributes, environmental attributes, characteristics, and manner of injuries are essential for planning and operationalizing a comprehensive injury prevention program. M S Ramaiah Medical College hospital has been an active participant in injury surveillance program in Bangalore and this study was conducted in the hospital to study the pattern of injuries in M S R Hospitals since past 1 year. This information will be utilized to improve the quality of health care and conceptualize an injury prevention capsule so as to make the care comprehensive.

## Materials and Methods

This hospital-based cross-sectional study was conducted in M S Ramaiah Medical College Teaching Hospital and M S Ramaiah Memorial Hospital, Bangalore. All patients seeking health care for injury at MSR hospitals over the past 1 year April 2007-March 2008 were included for the study and interviewed using a pretested semi-structured proforma. The patients admitted with delayed complications of road traffic injuries were excluded. Information was collected from the patients/attendants regarding the various components of injury by the casualty medical officers, post-graduates, and house surgeons. The data collected was analyzed using SPSS version 16 to compile the descriptive and analytical statistics.

## Results and Discussions

A total of 1055 cases of injures sought health care during the study period. The mean age of the injured was 31.3 years (SD: 14.7 years). 745 (74%) of the injured subjects were between 15 and 44 years and this is almost similar to figures in Karnataka where three-fourth of the injuries occur in this age group.(3) 76.4% (806) of the injured subjects were males and 88.4% of the injured were literates.

In the present study, the highest number of patients seeking health care in our hospital were males and more so the younger ages between 15 and 44 years who are the productive age group. A similar pattern was observed in other studies, in Delhi it was observed that the accident rates were 4.9 times higher in males than in females.(4) Another study reports as high as 80% of the victims were males.(5) The gender difference is probably related to both exposure due to increased mobility and risk taking behavior.

### Injury details

72.1% of injuries occurred within the Bangalore city. Road traffic injuries continue to predominate the injury scene as 694 (65.8%) were injured on the road, 200 (19%) were injured at home, and 95 (9%) were injured at workplace. Poisoning was cause of injury among 123 (12.3%) followed by falls 68 (6.4%) and assault 62 (5.9%). In other studies in Bangalore, it was observed that 45% of all injuries were due to road traffic injuries, 10% due to poisoning, 17% due to burns, and 7% due to falls.(2)

794 (75.3%) of the injuries were unintentional, 66 (6.3%) were intentional assault, 135 (12.8%) were self-harm injuries and among the rest the reason was not known. 62 (5.9%) of the injured gave history of alcohol consumption when injured.

### Road traffic injury details

457 (65.8 %) of the road traffic injuries occurred in city/municipal roads, 105 (10%) occurred in the highway, and around 54 (5.1%) occurred in rural roads. Among 694 road traffic accident patients, 304 (43.8%) were two-wheeler riders, 87 (12.5%) two-wheeler pillion, 28 (4.03%) three-wheeler drivers, 43 (6.1%) car occupants, 19 (2.7%) car drivers, and 129 (18.5%) were pedestrians. It was observed that nearly 55% of the road traffic injuries involved two wheelers among whom only 198 (50%) used helmets. Thus any attempt to prevent and control RTI, this major risk group has to be the target for focused intervention. Among the 62 car drivers/occupants injured, only 12 (19.3%) had used seat belts.

### Injury severity, management, and outcome

Most common site of injury was head 375 (35.5%) followed by lower limbs 342 (32.4). 111(10.5%) had severe injuries (massive internal or external bleeding and vital signs are unstable which requires admission and immediate aggressive management), 684 (64.8%)had moderate injuries (fractures, external or internal bleeding, large open wound where there is suspected injury to internal organs, vitals are stable which requires hospital admission or stay in casualty for more than 6 h), and 199 (18.9%) had mild injuries (abrasions, lacerations, etc. where patient does not require hospital admission and can be sent home after observation).

69 (10%) of the RTI and 10% of all unintentional injuries were severe. 836 (79.2%) of the patients were conscious at time of seeking health care, 90 (8.5%) were semi-conscious. 78 (7.4%) unconscious and 47 (4.5%) were brought dead. Among the injured, 702 (66.5%) were admitted for medical/surgical care, 77 (7.3%) were treated in emergency room and referred to another hospital, and 149 (14.1%) were treated in emergency room and sent home. At the end of casualty management, 557 (52.8 %) improved, 44 (4.2%) condition worsened, and 17 (1.6%) died.

## Conclusions

Road traffic injury continues to predominate the type of injuries seeking health care since past 1 year in MSR hospitals followed by poisoning and falls. Two wheelers were commonly involved in RTI with riders being injured more than pillion. RTI was more among pedestrians as compared to four wheelers implying the immediate need to focus on pedestrian safety. This pattern seen in MSR hospitals is in contrast to other findings in Bangalore where majority of the RTI injured were pedestrians and not two wheelers.(3)

In spite of an existing legislation on compulsory use of helmets, only half of two wheeler users complied with the legislation and even smaller percentage of four wheeler users had worn seat belts. Thus there is a need to focus on safety education and strict enforcement of the existing legislations as an immediate follow up to the advocacy measure.

A majority of the injuries were moderate in severity with head being the most common site to get injured. Most of the cases were managed and improved in our hospital with very small percentage of referrals.

Thus, to conclude from the present study, it was found that road traffic injury was the most common among the patients seeking health care for injuries in our hospital. Lacerations, head injuries, and basically moderate type of injuries were observed. Among the victims of road traffic injuries, two wheelers and pedestrians were commonly involved and more commonly on city roads rather than highways. Injury victims belonged to the young and productive age group probably because of increase in the exposure and may be attributed to risk-taking behavior.

Safety education regarding road safety should be imparted especially to all victims, relatives, and general public to make the care comprehensive. Students in schools and colleges should also be the target for intense safety education.
